# Nonuniform Internal Structure of Fibrin Fibers: Protein Density and Bond Density Strongly Decrease with Increasing Diameter

**DOI:** 10.1155/2017/6385628

**Published:** 2017-10-10

**Authors:** Wei Li, Justin Sigley, Stephen R. Baker, Christine C. Helms, Mary T. Kinney, Marlien Pieters, Peter H. Brubaker, Roger Cubcciotti, Martin Guthold

**Affiliations:** ^1^Department of Physics, Wake Forest University, Winston-Salem, NC 27109, USA; ^2^Leeds Institute of Cardiovascular & Metabolic Medicine, The LIGHT Laboratories, University of Leeds, Clarendon Way, Leeds LS2 9NL, UK; ^3^Department of Physics, University of Richmond, Richmond, VA 23173, USA; ^4^Centre of Excellence for Nutrition, North-West University, Potchefstroom, South Africa; ^5^Department of Health & Exercise Science, Wake Forest University, Winston-Salem, NC 27109, USA; ^6^NanoMedica, LLC, Biotech Place, 575 Patterson Ave, Winston-Salem, NC 27101, USA

## Abstract

The major structural component of a blood clot is a meshwork of fibrin fibers. It has long been thought that the internal structure of fibrin fibers is homogeneous; that is, the protein density and the bond density between protofibrils are uniform and do not depend on fiber diameter. We performed experiments to investigate the internal structure of fibrin fibers. We formed fibrin fibers with fluorescently labeled fibrinogen and determined the light intensity of a fiber, *I*, as a function of fiber diameter, *D*. The intensity and, thus, the total number of fibrin molecules in a cross-section scaled as *D*^1.4^. This means that the protein density (fibrin per cross-sectional area), *ρ*_*p*_, is not homogeneous but instead strongly decreases with fiber diameter as *D*^−0.6^. Thinner fibers are denser than thicker fibers. We also determined Young's modulus, *Y*, as a function of fiber diameter. *Y* decreased strongly with increasing *D*; *Y* scaled as *D*^−1.5^. This implies that the bond density, *ρ*_*b*_, also scales as *D*^−1.5^. Thinner fibers are stiffer than thicker fibers. Our data suggest that fibrin fibers have a dense, well-connected core and a sparse, loosely connected periphery. In contrast, electrospun fibrinogen fibers, used as a control, have a homogeneous cross-section.

## 1. Introduction

### 1.1. Fibrinogen

Fibrinogen is a key protein in blood, which upon activation by thrombin polymerizes into a meshwork of fibrin fibers. Fibrin fibers form the major structural component of blood clots. In hemostasis, they stem blood flow in the event of injury and trauma, and they are involved in the initiation of wound healing [1]. In thrombotic disease, aberrant formation of blood clots is the underlying pathology of such diseases as myocardial infarction, ischemic strokes, deep vein thrombosis, and pulmonary embolisms. Fibrinogen is a 340-kDa glycoprotein, with an elongated, trinodular shape; it is 45 nm in length and 4.5 nm in diameter. It is composed of two distal D regions and one central E region, which are connected by two triple-helical coiled coils [2–4].

### 1.2. Protofibril Formation

The first step in fibrin fiber formation is the formation of protofibrils. It is initiated as thrombin proteolytically removes fibrinopeptide A from the central E region of fibrinogen, thus converting fibrinogen to desA fibrin. Knob “A”, which remains after cleavage of fibrinopeptide A, fits into hole “a”, located in the terminal D region. Fibrin monomers assemble via these A:a interactions in a half-staggered fashion into double-stranded protofibrils. The interactions between abutting D interfaces, called D:D interactions, are also important for protofibril assembly. It is well accepted in the field that the key interactions for protofibril assembly are the A:a and D:D interactions. If these interactions are blocked, protofibril formation is severely impaired, which is not the case for any of the other interactions.

### 1.3. Lateral Aggregation

The two-stranded protofibrils then assemble laterally (radially) into mature fibrin fibers [5–7]. However, the interactions for this lateral assembly are much less understood, and the internal arrangement of protofibrils within a mature fiber is not clear. Some studies suggest a relatively regular internal structure, in which the protofibril is packed in a semicrystalline fashion [6, 8] while others suggest that it is packed randomly [9–11]. A commonly used method to investigate the properties and the structure of fibrin clots is turbidimetry and light scattering, which can provide information about fiber size [12, 13] and fiber (protein) density [14, 15]. There are numerous papers about the mechanical properties and structures of a whole blood clot [16, 17]. However, the literature on the emerging field of single fibrin fiber mechanical properties is more limited [18–27]. Moreover, scattering techniques which draw conclusions about single fibers take data that are averaged over many fibers, and then information about single fibers is extracted; in other words, the information about single fibers is indirect [28]. If there are significant differences in single fibers, that difference may be lost in averaging methods. When measuring single fibers, differences between individual fibers can be resolved.

### 1.4. Fibrin Fiber Lateral Structure

The lateral aggregation of fibrin fibers is poorly understood. In the past, most models assumed a uniform cross-sectional density [6, 8]. Recently there has been some evidence that the internal structure is not homogeneous [28, 29]. Protein density is a good indicator of the fiber structure. Neutron scattering and light scattering are most frequently used to indirectly determine protein density. These techniques have been used to show that protein content inside a fibrin density is only 20–30% (70–80% solvent) (some theoretical studies tried to explain this phenomenon [30, 31]) and the lateral structure of fibrin fibers may be fractal [28]. Similar results were found by high-resolution atomic force microscope (AFM), as it was observed that molecule packing inside the fiber might be denser and tighter than that on the surface [29, 32]. Early rupture force measurements on dry fibrin fibers suggested that the cross-section of fibrin fibers may have a fractal dimension of 1.3 [9].

In the presented work, we used fluorescently labeled fibrinogen to obtain a relationship between fiber light intensity, *I*, which is proportional to the number of monomers in a fiber cross-section, and fiber diameter, *D*. We found *I* ∝ *D*^1.4^, suggesting a denser core and a less dense periphery. For a fiber with uniform density, the number of fibrin molecules would increase as the square of the diameter, *D*^2^.

In addition to the fibrin and protofibril packing inside a fiber, the bonds involved in lateral aggregation are also not well understood. Bonds are important because they hold the fiber together. In the past, there has been no method to determine bond density inside a fiber. The stretching force of a single fiber is proportional to the number of bonds inside the fiber. For a fiber with homogeneous bond density, the stretching force would increase as *D*^2^ (since the cross-sectional area of a fiber increases as *D*^2^). However, our data show the stretching force, *F* ∝ *D*^0.6^; that is, it increases with a significantly lower power than *D*^2^. This agrees with our proposed model that the inside of a fibrin fiber is not homogeneous.

This relationship was found for fibrin fibers formed from diverse sources: purified fibrinogen and plasma from white older males, white middle-aged males, and black middle-aged females with and without diabetes. Since this relationship was found for all samples, it appears to be a general property of fibrin fibers, regardless of the source.

Combining our light intensity and force data, we propose a novel model for the internal structure of fibrin fibers. In this model, they have a dense core of well-connected protofibrils that becomes less dense and less well connected as more protofibrils aggregate onto the outside of the fiber.

## 2. Materials and Methods

### 2.1. Plasma Collection

Blood samples were from several different population groups. Blood was collected from healthy white males from two different subgroups: five healthy middle-aged individuals (40–50 years old) and five healthy older individuals (>70 years old). Blood samples were collected into citrated tubes and then centrifuged in a Beckman model TJ-6 centrifuge at 3700 rpm (2000*g*) for 15 minutes at room temperature to remove cells and large particles; the remaining plasma was stored at −80°C until further use. These samples were covered under WFU IRB protocol 00012738.

Blood was also collected from black, female South Africans from three different subgroups: five controlled diabetic patients (Type II Diabetes) (receiving insulin treatment), five uncontrolled diabetic patients (Type II Diabetes), and a control group of five healthy (nondiabetic) subjects [18]. These samples were a random subset of a larger sample [33]. Blood from these groups was centrifuged at 2000*g* at 4°C for 15 minutes. The remaining plasma was stored at −80 until further use. All subjects signed informed consent and ethical approval was obtained from the ethics committees of both the University of Pretoria and the North-West University, South Africa. [33].

### 2.2. Striated Substrate Preparation

The striated substrates were prepared as previously reported [18–22]. Briefly, a drop of optical adhesive (NOA-81, Norland Products, Cranbury, NJ) was placed on top of a coverslip. A rectangular polydimethylsiloxane (PDMS) stamp was then pressed into the optical glue to form a striated substrate with 6.5 *μ*m wide ridges and 13.5 *μ*m wide grooves. The optical glue was then cured under 365 nm UV light (UVP 3UV transilluminator, Upland, CA) for 1.5 minutes, at which point it was ready to be used.

### 2.3. Formation of Electrospun Fibrinogen Fibers and Fibrin Fibers

#### 2.3.1. Electrospun Fibrinogen Fibers

100 mg/ml lyophilized bovine fibrinogen (Sigma-Aldrich Chemical Co.) was prepared with a solution of 9-part 1,1,1,3,3,3-hexafluoro-2-propanol (HFP, Sigma-Aldrich) and 1-part minimum essential medium (MEM, 10x MEM, Gibco, Invitrogen cell culture). 3.17 mg* Rhodamine 6G *fluorophore (Eastmen Kodak, Rochester, NY; molecular weight 479 g/mol) was then added to the mixture to get a final ratio of 15 fluorophores per fibrinogen molecule. The electrospun fibrinogen fibers were spun onto a cover glass slide (number 1.0, 24 mm × 60 mm, Fisherbrand, Pittsburgh, PA) as described in [34].

#### 2.3.2. Fibrin Fibers from Plasma Samples

Samples were prepared as described in [18]. All chemicals were from Sigma-Aldrich unless otherwise noted. Fibrin fibers were formed from the human, platelet-poor plasma samples described above. 2 *μ*l of 0.1 NIH units/ml thrombin was added to an 18 *μ*l mixture of plasma and 20 mM CaCl_2_ that was first pipetted onto the striated substrate. The sample was kept in a wet atmosphere at room temperature for one hour. After that, the top layer of the fibrin clot was gently rinsed away using calcium free fibrin buffer (140 mM NaCl, 10 mM, Hepes, pH 7.4), and 20 nm carboxyl coated fluorospheres (Invitrogen, Carlsbad, CA) were added to the substrate. The sample was incubated in fibrin buffer before use.

#### 2.3.3. Fibrin Fibers from Purified Fibrinogen and Fluorescently Labeled Fibrinogen

Fibrin fibers were formed from purified unlabeled fibrinogen (Enzyme Research Laboratories, South Bend, IN) and purified fibrinogen that was labeled with Alexa-546 fluorophore (about 15 fluorophores per fibrinogen monomer; Life Technologies, Grand Island, NY). 18 *μ*l of fibrinogen solution (13.5 *μ*l nonlabeled and 4.5 *μ*l fluorescently labeled fibrinogen, both at 1.5 mg/ml) and 2 *μ*l of 0.1 NIH units/ml thrombin were pipetted onto a cover glass slide (number 1.0, 24 mm × 60 mm, Fisherbrand, Pittsburgh, PA). The sample was kept in a wet atmosphere at room temperature for one hour. After this period, the top layer of the fibrin clot was gently rinsed away with fibrin buffer, and the sample was kept in fibrin buffer (140 mM NaCl, 10 mM Hepes, and 5 mM CaCl_2_, pH 7.4).

We performed turbidity experiments with unlabeled fibrinogen and 3 : 1, 1 : 1, and 1 : 3 ratios of unlabeled fibrinogen to Alexa-546-labeled fibrinogen (see Supplementary Figure S1 in the Supplementary Material available online at https://doi.org/10.1155/2017/6385628). Our preparation of Alexa-546-labeled fibrinogen had about 15 dye molecules per fibrinogen molecule; Alexa-Fluor-546 NHS Ester has a molecular weight of 1160 g/mole. The turbidity experiments were performed with the same buffer conditions as the fluorescence intensity experiments (same salt concentrations and pH, same fibrinogen concentrations and ratios). The experiments showed that Alexa-546-labeled fibrinogen does impair fibrin fiber formation in a dose-dependent manner. Specifically, the slope is less steep and the maximum absorption is lower. The lowest dose of labeled fibrinogen—the 3 : 1 ratio we used in the fluorescence intensity experiments—still has some impairment on fibrin fiber formation. However, we believe these fibers are still a reasonably good representation of natural fibrin fibers. This assessment is based on the appearance of the fibers in AFM and fluorescence images and on the overall shape of the turbidity curve, which looks normal. In our intensity experiments, we had to find a compromise between enough labeling, so that the fibers would be clearly visible in the fluorescence images, and too much labeling, which impairs fibrin fiber formation. We believe the 3 : 1 ratio is a good compromise between these two competing requirements.

#### 2.3.4. Fibrin Fibers from Unlabeled Purified Fibrinogen for Force Measurement

Fibrin fibers were formed as described above, except that they were formed from nonfluorescently labeled, purified fibrinogen on a striated substrate, and labeled with 20 nm carboxyl coated fluorospheres after polymerization (Invitrogen, Carlsbad, CA) as described above.

### 2.4. Fluorescence Microscopy (Optical Microscopy)

We used an inverted fluorescence microscope (Axiovert 200 or Observer D, Zeiss, Göttingen, Germany) in these experiments. Fluorescence images were taken with a 40x lens (NA of 0.7), with an excitation source from a short arc mercury lamp (Osram HBO 103W/2, Atlanta Light Bulbs Co., Atlanta, GA). The exposure time was 200 ms. The field of view (image size on computer) was 180 *μ*m × 180 *μ*m. In order to get the same photobleaching for all the fibers in a particular image, fluorescence images of each area were taken only once, under the same conditions. Fluorescence images were collected with a Hamamatsu EM-CCD C9100 camera (Hamamatsu Photonics, KK, Japan) with IPLab software (Scanalytics, Fairfax, VA).

### 2.5. Fibrin Fiber Measurements and Manipulation

#### 2.5.1. Fluorescence Intensity Measurement

**Figure 1:**
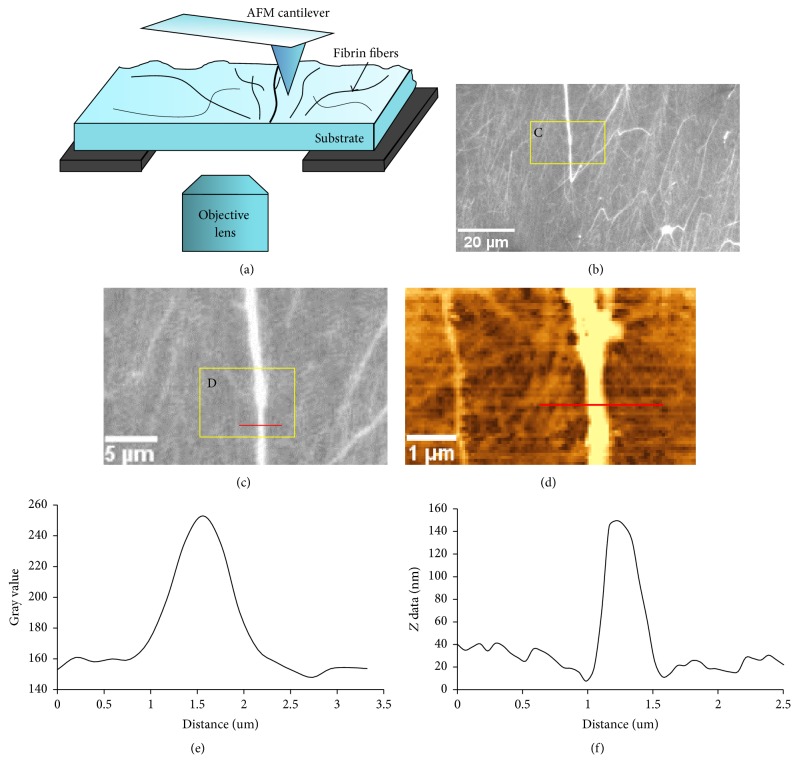
Experimental setup of fluorescence intensity measurement. (a) Schematic lateral view of setup. The AFM is above the sample, and the objective lens of the inverted fluorescence microscope is below the sample. ((b), (c)) Fluorescence images of fibrin fibers. (d) AFM topography image of the same region (yellow box) of fibrin fibers as in (c). (e) Fluorescence intensity distribution of the cross-section (along the red line in (c)) of a single fiber; total fluorescence intensity of this fiber corresponds to the area under this curve. (f) Height distribution of the cross-section along the red line of the fiber in (d), which is the same fiber as in (c). Even though the fiber in (c) looks very bright, it was far below saturation as the gray value limit is 16384 (2^14^).

Fibrin fibers (or electrospun fibrinogen fibers) were formed on a coverslip and fluorescence images of the fibers were taken immediately after illuminating the fibers to limit photobleaching as much as possible. Since the diameter of fibrin fibers is below the resolution limit of light microscopy, the AFM was used to obtain images of the same location as the fluorescence image (Figure 1). Assuming a cylindrical fiber, fiber height as determined from AFM topography images was taken as the fiber diameter (fiber width was not used because it is significantly exaggerated in AFM due to the tip broadening effect). ImageJ software (https://imagej.nih.gov/ij/) was used to determine the light intensity of the cross-section of single fibers. The lowest point was used as the baseline to normalize intensity and the total intensity was obtained by summing the intensities of each pixel.

#### 2.5.2. AFM Imaging of Fibrin Fibers

All fibrin fibers were imaged in buffer (same buffers as described above under fibrin fiber preparation), using a combined atomic force microscope (AFM) (Topometrix Explorer, Veeco Instruments, Woodbury, NY) and inverted fluorescence microscope (Axiovert 200 or Observer D, Zeiss, Göttingen, Germany). The fiber sample was placed between the AFM and optical microscope using a customized stage which allows the sample to be moved independently of either microscope [18–22]. Fibers were imaged in tapping mode with any one of the three cantilevers of AFM probe CSC-38 (MikroMasch, Wilsonville, OR) or an equivalent AFM probe (similar *k* and *f*). The spring constant, *k*, ranged from 0.03 N/m to 0.09 N/m, and the resonance frequency, *f*, ranged from 10 kHz to 20 kHz. Fibers were typically imaged at a 50% set point (50% of maximum free amplitude); the set point was adjusted so that the probe exerted the smallest possible normal force on the sample, while still making good contact with the surface. Feedback gains were adjusted as high as possible, without causing ringing. The fiber diameter, *D*, was determined by AFM imaging the fiber either on top of the ridge adjacent to where the fiber was manipulated (for the force measurements) or on the glass slide (for the fluorescence intensity measurements). The fiber cross-section was calculated assuming a cylindrical cross-section.

#### 2.5.3. Force Data Measurement

**Figure 2:**
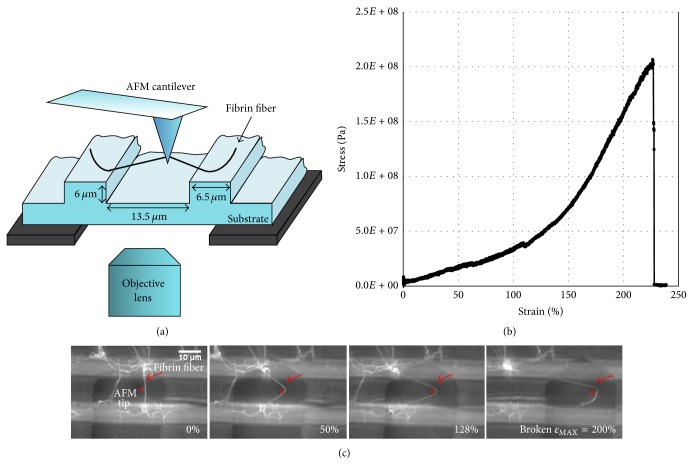
Experimental setup for force measurement. (a) Schematic lateral view of setup. (b) Representative single fibrin fiber stress versus strain plot. The fiber broke at a strain of 225%. The modulus corresponds to the slope of this plot. We used the initial slope, before strain hardening at about 100% strain. (c) Fluorescence images of a fiber being stretched and broken. The large dark object is the AFM cantilever and the AFM tip is marked by an asterisk. The fiber in (c) broke at a strain of 200%. Figures 2(a) and 2(c) are adopted from [18].

Fibrin fiber mechanical properties were determined using a combined fluorescence microscope and atomic force microscopy (AFM) technique, as described before [18–20, 22]. Briefly, as shown in Figure 2, fibers were formed on the striated substrate. The AFM (Topometrix Explorer, Veeco Instruments, Woodbury, NY) tip is located above the sample for manipulation, while the inverted fluorescence microscope (Axiovert 200 or Observer D, Zeiss, Göttingen, Germany) can image the sample from below.

Single fibers, suspended over the 13.5 *μ*m grooves in the striated substrate, were laterally pulled causing the AFM cantilever to torque. NanoManipulator software (3rd Tech, Chapel Hill, NC) provided precise control of the AFM tip and collected force and position data during fiber manipulations. Fiber diameters were measured on the ridges of the striated substrate using the AFM tapping mode (and assuming a cylindrical fiber). From these data, the actual force on the fiber was calculated; and stress and strain were determined as *σ* = *F*/*A* and *ε* = Δ*L*/*L*_init_, where *A* is the fiber cross-sectional area, *L*_init_ is the initial length of the fiber, and Δ*L* is the change in fiber length. Young's (elastic) modulus, *Y*, is then calculated via *Y* = *σ*/*ε* from the initial part of the stress-strain curve. Possible fiber compression effects where the AFM probe contacts the fiber were ignored. Since the fiber is 13.5 *μ*m long and the tip contacts the fiber over a length of only about 500 nm (the very end of the AFM tip is used for manipulations), this contact length is less than 4% of the entire fiber length.

### 2.6. Statistical Analysis

The significant difference between two groups was determined by using a two-tailed *t*-test. The significant difference between two slopes was calculated by analysis of covariance (ANCOVA).

## 3. Results

The goal of our work was to investigate the internal structure of fibrin fibers; in particular we aimed to determine how the number of fibrin monomers and the number of bonds inside a fibrin fiber vary as a function of fiber diameter. These data will provide insights into the structural arrangement of fibrin monomers and bonds inside a fibrin fiber. We used two types of experiments to determine these quantities. (1) Fluorescence intensity measurements of single fibers formed from fluorescently labeled fibrinogen: these data provide information on the number of fibrin monomers in a fiber. (2) Stretching force measurements on single fibers: these data provide information on the number of bonds in a fibrin fiber.

### 3.1. Fluorescence Intensity as a Function of Fiber Diameter

In these experiments, fibrin fibers were formed on a flat coverslip from fibrinogen that was labeled with Alexa-546. The fiber diameter, which is beyond the resolution limit of light microscopy, was determined from an AFM topography image of the same area (Figure 1). A cylindrical fiber shape was assumed and the fiber height was used as the diameter of the fiber. We used the height because it can be determined accurately, with nanometer precision. In AFM imaging, the height of the sample can sometimes be compressed by the force exerted on the sample by the AFM tip. However, this effect is typically small. Comparing our diameter values to values obtained by other techniques suggests our height measurements are correct and that it is reasonable to assume the fibers are cylindrical. In particular, our diameter values determined from the height of the fibers agree with the values obtained by SEM [18, 33]. Our diameter values also generally agree with the diameter values obtained by light scattering [28]. The lateral dimensions in atomic force microscopy images are always exaggerated by the tip broadening effect, as the width of the AFM probe gets added to the width of the sample [36]. Therefore, the fiber width in AFM images is not an accurate representation of the fiber diameter.

Photobleaching was minimized by having the shortest possible exposure times and by taking images as rapidly as possible. Fluorescence images of each area were taken only once, under the same conditions, so that residual photobleaching was the same for all fibers in a particular image. Moreover, to average out small fluctuations, we analyzed four closely spaced cross-sections of each fiber (see Figure 1) and took the average. We did the same four-measurement averaging procedure to determine the diameter of each fiber from the AFM scans.

### 3.2. Dry Electrospun Fibrinogen Fiber (Mixed with* Rhodamine 6G*)

**Figure 3:**
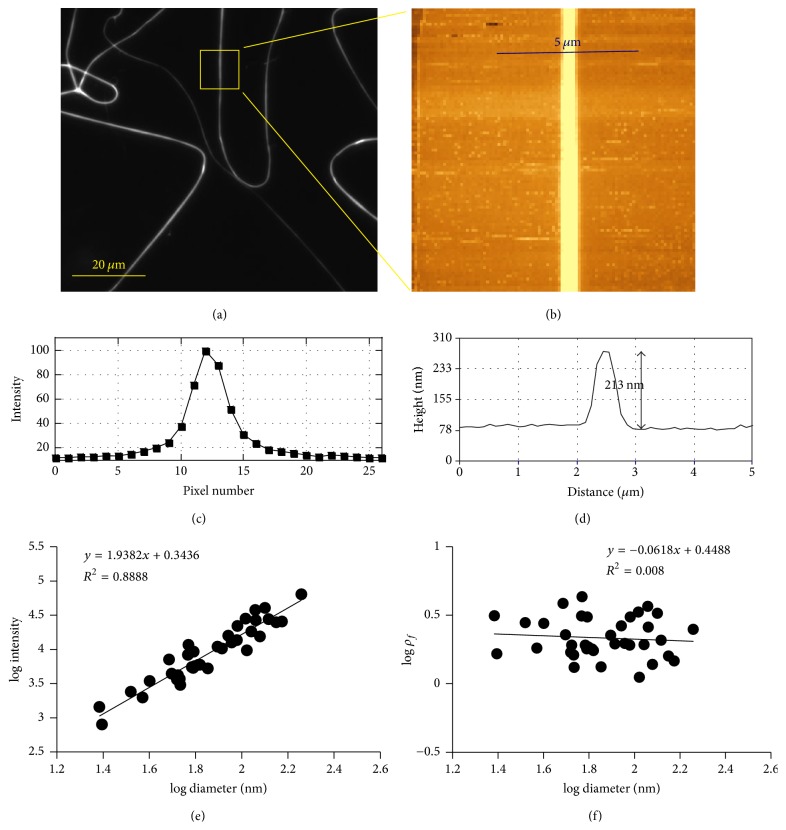
Fluorescence intensity of electrospun fibrinogen fiber cross-section as a function of diameter. (a) Fluorescence image (subsection of 180 *μ*m × 180 *μ*m field of view) of electrospun fibrinogen fibers. (b) AFM image of the fiber in the yellow box. (c) Fluorescence intensity of the fiber in the yellow box along the blue line drawn in (b). One pixel is 200 nm (40x objective lens). (d) Height measurement along the indicated 5 *μ*m long blue line in (b). (e) The slope of the relationship between light intensity and fiber diameter is 1.9 on a log-log scale, close to the value of 2.0 expected for a homogenous fluorophore distribution within a cross-section. (f) Fluorophore density *ρ*_*f*_ = *N*_*f*_/*A* is independent of fiber diameter, as would be expected for a homogeneous fiber. Each data point represents four measurements.

As a control experiment, we first determined the fluorescence intensity of electrospun fibers (Figure 3). Electrospun fibrinogen fibers have about the same diameter as fibrin fibers, on the order of 100 nm [35]. Dye was mixed into the spinning solution. For a homogeneous cross-section with an evenly distributed dye, the fluorescence intensity should increase as *D*^2^, since the cross-sectional area of a fiber (assuming it is cylindrical) is (*π*/4)*D*^2^. The molar ratio of fluorophore to fibrinogen monomer in the spinning solution was 15 : 1.

In this control experiment, we found that the total light intensity, *I*, increases with increasing diameter as *I* ∝ *D*^1.9±0.1^, which means that the number of fluorophores, *N*_*f*_, also increases as *N*_*f*_ ∝ *D*^1.9±0.1^ (Figure 3(e)). This experiment was performed in triplicate (for images and additional data, see Supplementary Figure S2). If we assume a circular cross-section of the electrospun fiber, *A* = (*π*/4)*D*^2^, the fluorophore density *ρ*_*f*_ = *N*_*f*_/*A* is constant, as would be expected for a homogeneous fiber with an even distribution of fluorophores (Figure 3(f)).

### 3.3. Wet Fibrin Fiber Labeled with Alexa-546 Fluorophore

**Figure 4:**
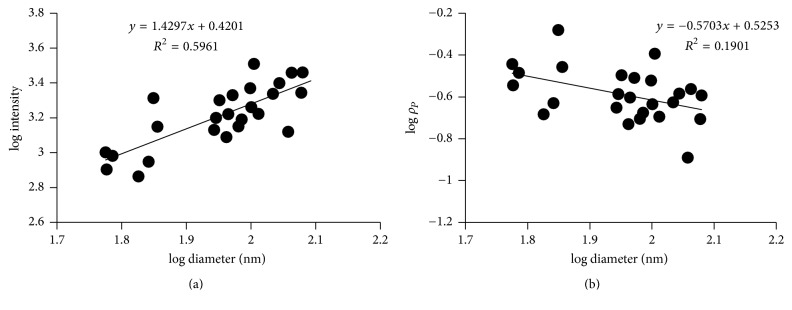
Fluorescence intensity of fibrin fibers (in buffer) as a function of diameter. (a) The slope of the relationship between light intensity and fiber diameter is 1.4 on a log-log scale. (b) The slope of the relationship between protein density and fiber diameter is about −0.6 on a log-log scale. Each data point represents four measurements.

A 3 : 1 mixture of unlabeled fibrinogen and fibrinogen that was labeled with 15 Alexa-546 fluorophores per fibrinogen monomer was used to form fibrin fibers on a flat glass substrate. For these fibrin fibers, fluorescence intensity also increased with increasing diameter; however it only increases as *I* ∝ *D*^1.4±0.2^ (Figure 4(a)). Since the total fluorescence intensity, *I*, of a fiber cross-section is proportional to the number of monomers in a cross-section, *N*_*m*_, this implies that *N*_*m*_ ∝ *D*^1.4±0.2^. This is a much lower exponential power than 2, which was seen in the electrospun control fibers and which would be expected for homogeneous fibers (these experiments were carried out in triplicate; for other plots, see Supplementary Figure S3). To obtain the diameter dependence of the fibrin density (protein density), we divide by the cross-sectional area (assuming a cylindrical cross-section, *A* = (*π*/4)*D*^2^). Thus, the protein density scales as *ρ*_*p*_ ∝ *D*^−0.6^; it decreases strongly with increasing diameter (Figure 4(b)). This means that thinner fibers have a higher protein density than thicker fibers. It suggests that the cross-section of a fibrin fiber is not homogeneous and that fibrin fibers have a higher density in the core and a lower density at the periphery.

Dry fluorescently labeled fibrin fibers showed a similar relationship, as the intensity, *I*, varied as *I* ∝ *D*^1.21±0.14^ (details in Supplementary Figure S4).

### 3.4. Fiber Modulus as a Function of Fiber Diameter

The number (and strength) of parallel, longitudinal bonds inside a fiber is proportional to the force that is required to stretch the fiber. Therefore, determining the stretching force as a function of diameter can provide information on the bond density of a fiber cross-section. Before stretching a fiber, we first used the AFM in imaging mode to determine the diameter of the fiber (on the anchoring ridge). We then used the AFM tip to pull the same fiber to obtain a stress-strain curve. This procedure is described in detail in [20, 35]. Young's modulus (stiffness), *Y*, of the fiber corresponds to the slope of the stress-strain curve. The fibrin fibers in these force measurements were formed from plasma from various groups (see methods) and also from purified fibrinogen.

In all of our force experiments, for every sample, we took at least 20 measurements, so there are at least 200 data points in each group. The three different groups are plasma samples from white males, from black females, and from purified fibrinogen. We saw very similar relationships between the modulus and fiber diameter for fibers from all groups. The modulus, *Y*, decreased strongly as fiber diameter increases with an exponential power of about −1.5; that is, *Y* ∝ *D*^−1.5^. This appears to be a general property of fibrin fibers, since we saw a similar relationship for all groups.

**Figure 5:**
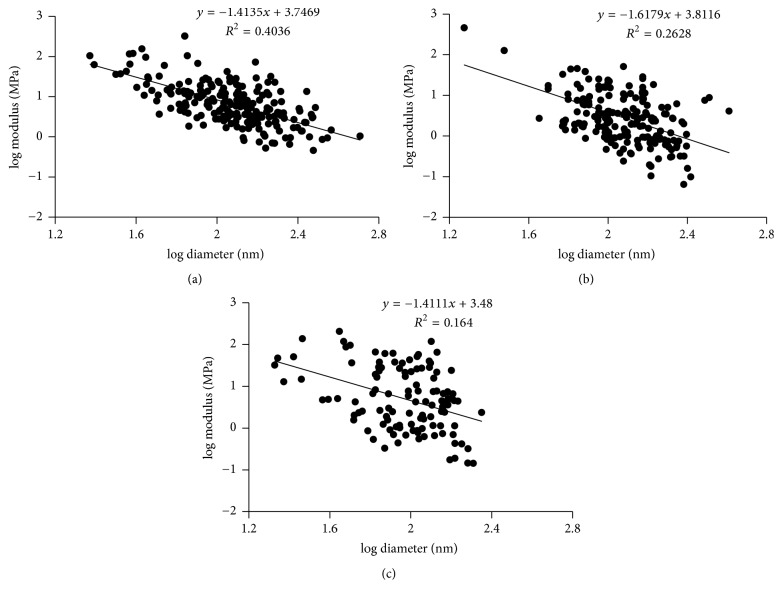
Fibrin fiber modulus as a function of diameter. (a) For plasma samples from white males, the slope was −1.4 ± 0.1 on a log-log scale. (b) For plasma samples from black females, the slope was −1.6 ± 0.2. (c) For samples from purified fibrinogen, the slope was −1.4 ± 0.3. Figures 5(b) and 5(c) are adopted from [18].

Specifically, for plasma samples from white males, the slope of this relationship was −1.4 ± 0.1 on a log-log scale, while the slope for plasma samples from black females was −1.6 ± 0.2. The slope for samples from purified fibrinogen was −1.4 ± 0.3 (Figure 5).

 Young's modulus may be related to the bond density, *ρ*_*b*_, inside a fiber. To make this connection, we assume that the lateral bonds will also carry (transmit) some of the longitudinal stress, when a fiber is strained. This is a reasonable assumption for current models of fibrin fibers, in which the protofibrils are connected to each other by lateral bonds. There is evidence that these lateral bonds include linked *α*C regions and perhaps B:b interactions [23, 24]. For the longitudinal stress to be transmitted from one protofibril to the next, these lateral bonds would have to carry (transmit) the longitudinal stress. In other words, the lateral bonds connect the protofibrils. This would put the lateral bonds in a series connection with the protofibrils. In a longitudinal pull, since the protofibrils and the *α*C regions are in series, they would both experience the applied force. This means that the *α*C regions would unravel and the fibrin monomers could partially unfold (*α*-helical to *β*-sheet transition of the *α*-helical coiled coils and some other domains could unfold).

In this fiber model, the modulus is then proportional to the bond density, *ρ*_*b*_, that is, the number (and strength) of bonds per unit cross-sectional area. For example, a doubling of the bonds per unit area would result in a doubling of the modulus. Since *Y* ∝ *D*^−1.5^, the bond density, *ρ*_*b*_, also scales as *ρ*_*b*_ ∝ *D*^−1.5^. This means that the bond density decreases drastically with increasing diameter, and thin fibers have a much higher bond density than thick fibers. Since fibers grow from the inside out, by adding more protofibrils to the outside, this finding also implies that the bond density in a given fibrin fiber is not uniform. Our data imply that the bond density strongly decreases as the fiber diameter increases—fibrin fibers have a much higher bond density in the center than at the periphery.

## 4. Discussion

In our study, we found that the light intensity, *I*, which is proportional to the number of fibrin molecules in a fiber depends on fiber diameter, *D*, as *I* ∝ *D*^1.4^ for wet fibrin fibers. This implies that the protein density, which is proportional to the intensity divided by the cross-sectional area, *A* = (*π*/4)*D*^2^, decreases as *ρ*_*p*_ ∝ *D*^−0.6^. That is, fibrin fibers have a higher protein density in the center than at the periphery. In electrospun fibrinogen fibers, which were used as control fibers with a homogeneous cross-section, the light intensity increased with fiber diameter *I* ∝ *D*^1.9^, close to the expected *D*^2^ for a constant density fiber. In the force measurements on different plasma and purified fibrinogen samples, the fiber modulus, *Y*, decreased with increasing fiber diameter *D* as *Y* ∝ *D*^−1.5^. This implies that the bond density (bonds per cross-section) also strongly decreases as *ρ*_*b*_ ∝ *D*^−1.5^, even stronger than the protein density, which decreased as *ρ*_*p*_ ∝ *D*^−0.6^. For a homogeneous fiber, the density would be independent of *D* (*ρ*_*b*_ ∝ *D*^0^).

### 4.1. Crosslinking

We did not inactivate FXIII or remove it from our samples. Thus, it is likely that residual FXIII in the plasma samples and the purified fibrinogen solution [37] was activated by thrombin and that the fibrin samples were, at least partially, crosslinked. The finding that all samples showed the same diameter dependence suggests that crosslinking does not have a significant effect on the internal arrangement of fibrin fibers. This is consistent with experiments showing that crosslinking does not have a significant effect on the internal arrangement of fibrin fibers, other than some slight reduction in fiber diameter [38].

### 4.2. Electrospun Fibrinogen Fibers

The main objective of the experiments with the electrospun fibers was to perform a control experiment with fluorescent nanofibers, in which the intensity would increase proportional to *D*^2^, as would be expected for a homogeneously labeled fiber. We used Rhodamine because it has a similar structure and spectrum as Alexa-546 and because it was soluble in the hexafluoropropanol solvent used for electrospinning.

### 4.3. Model of Fibrin Fiber Internal Structure

There is some evidence that the internal structure of fibrin fibers has some regularity and some crystallinity. In SEM images, a clear banding pattern with a spacing of 22.5 nm—half the length of a fibrin monomer—can often be seen [17, 39]. This fits well with the regular, half-staggered arrangement of the fibrin monomers in protofibrils and suggests a somewhat regular arrangement of protofibrils inside a fibrin fiber, as proposed in Yang et al.'s multibundle model [6]. However, this seemingly regular arrangement of protofibrils might have been partly induced by the vacuum conditions inside the SEM chamber. A similar, though less pronounced banding pattern with ~22.5 nm spacing, was also seen in some AFM images, though the pattern seems to disappear for larger fibers [29, 32].

Energy dispersive X-ray diffraction (EDAD) [8] and small angle X-ray scattering (SAXS) [28] were used to probe the internal structure of fibrin fibers. Peaks corresponding to lateral periodicity of 19 nm were broad and weak [28], indicating only weak ordering in the lateral (radial) direction. SAXS and light scattering data point to a fiber with a protein content of only 15% and a very porous cross-section that becomes increasingly porous as the diameter increases [28].

Most of these methods and techniques, like neutron scattering and light scattering [40, 41], only give indirect and averaged information about single fibrin fiber internal structure. Our experiments provide a more direct examination of fibrin fiber internal structure.

Our data are consistent with a model in which fibrin fibers do not have a homogeneous structure but rather a dense, well-connected core and a sparse, poorly connected periphery.

To explain our model, we should briefly discuss our data in the context of two simpler models. First, for a homogeneous cross-section of uniformly connected molecules, the intensity (number of molecules) and number of bonds would increase quadratically with diameter; the protein and bond density, and Young's modulus, would not depend on diameter. Second, for a model in which the cross-section looks like the spokes of a bicycle wheel, the intensity (number of molecules) and number of bonds would increase linearly with diameter; the protein and bond density, and Young's modulus, would decrease as *D*^−1^.

We observed that the relationship between the number of molecules and fiber diameter is *N*_*f*_ ∝ *D*^1.4^, thus, somewhere between a homogeneous fiber and the bicycle spokes model.

The situation for the bonds (connections that hold a fiber together) is somewhat different. We observed that the number of bonds only increases as *N*_*b*_ ∝ *D*^0.5^, thus, less than for the bicycle spokes model. This could be interpreted that fibrin fibers have a well-connected core but that connections drop off strongly toward the outside of the fiber.

As new technology developed in recent years, more detailed information about the internal structure of fibrin fiber was reported. Yeromonahos et al. used light scattering and small angle X-ray scattering measurements to investigate fibrin fiber internal structure [28]. Their data are consistent with a fractal fiber cross-section that would also have decreasing protein density with increasing diameter. High-resolution AFM imaging showed that thinner fibers are denser than thicker fibers and that molecular packing decreases as the fiber becomes thicker [29, 32]. Our data are also consistent with an earlier paper suggesting that a fibrin fiber cross-section has a fractal dimension of 1.3 [9].

**Figure 6:**
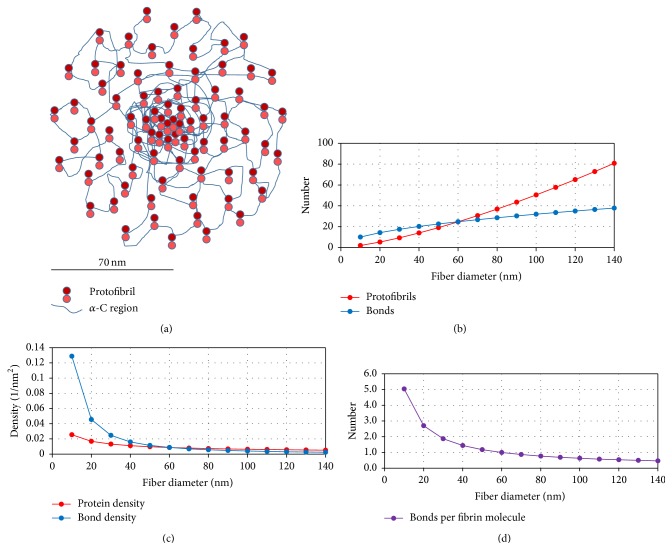
(a) Schematic cross-section of a hypothetical 140 nm diameter fiber with 81 protofibrils (approximately to scale). The fiber has a dense core of closely packed and well-connected protofibrils; the density then decreases toward the periphery. In this simplified depiction, protofibrils are connected by *α*C region interactions, ignoring other interactions (see main text). (b) Plot of the diameter dependence of the total number of protofibrils and the total number of bonds in a cross-section versus fiber diameter for the fiber depicted in (a). The number of protofibrils increases as *D*^1.4^ (same as the diameter dependence of fibrin monomers in a cross-section). The number of bonds increases as *D*^0.5^, and the molecules in the center of the fiber are assumed to have 5 bonds on average, which drops to less than 0.5 bonds for molecules at the periphery. These are only the bonds* between* protofibrils (interprotofibrillar bonds), and we do not count the classical intraprotofibrillar A:a and B:b bonds into this interprotofibrillar bond count. (c) Plot of the protein density and the bond density decreasing as *D*^−0.6^ and *D*^−1.5^, respectively. The schematic fiber in (a) corresponds to this protein density. (d) The number of interprotofibrillar bonds per monomer, starting with a hypothetical 5 bonds per monomer, dropping as *D*^−0.9^ (*D*^0.5^/*D*^1.4^). Fibrin molecules at the periphery have less than 0.5 interprotofibrillar bonds, on average. It should be noted that because protofibrils contain about 15 fibrin monomers, a protofibril, whose fibrin monomers have less than 0.5 bonds per monomer, can still be attached to a fiber. It should also be noted that the starting point of 5 interprotofibrillar bonds per monomer (in addition to the A:a and B:b interactions) is only an estimate. The estimate is based on (i) estimating how many interaction points the two large *α*C regions of a fibrin monomer might have and (ii) having about 0.5 bonds at the periphery.

Combining our light intensity and modulus data, we propose a possible internal structure model of a fibrin fiber, in which the fiber has a densely packed, well-connected core and a less dense, loosely connected periphery (Figure 6).

It has to be pointed out that this is a simplified model. The key feature, and only feature in this model that is supported by our data, is the power law of the decreasing protein and bond density. In the depicted model, the protofibrils have a parallel spatial arrangement along the fiber axis, and the protofibrils are held together only by the interactions of the *α*C region. There is good evidence that the *α*C region is important for lateral assembly [24, 42–46]. However, fibers can still form without this region [42]; thus, they are not absolutely required, and there are likely other lateral interactions. The model does not depict other possible interactions, such as the B:b interactions, which might be involved in lateral aggregation, and possible *D* region interactions [6]. It is also possible that the protofibrils, and the two fibrin strands within a protofibril, might not be arranged in the depicted parallel arrangement. In fact, Rocco et al. recently suggested the Y-ladder model for fibrin fiber assembly, based on coupled time-resolved X-ray and light scattering data [11]. In this model, fibrin monomers initially form (nonparallel) Y-ladder polymers, in which only one of the two A:a knob:hole interactions is engaged. Before the engagement of the second A:a bond between two fibrin units to form the classic double-stranded protofibrils, the Y-ladder polymers could allow the relatively frequent formation of branch points via off-axis binding of another activated monomer to the not-yet engaged a-site, leading to secondary chain growth. Therefore, many of these polymers may be interconnected before they coalesce to form thicker fibrin fibers. A possible consequence of the Y-ladder model is that the internal structure of fibrin fibers would be open, with some random internal branch points between fibrils. Fibers may contain some interconnected single-stranded fibrils and the “classical” double-stranded, half-staggered fibrils. Such an internal structure may also be consistent with the decreasing protein and bond density emerging from our data.

### 4.4. Possible General Assembly Mechanisms from Modeling

Computational modeling may also provide insights into the lateral assembly of fibrin fibers. A mechanism that may result in the observed nonuniform fiber with a denser core and a less dense periphery is diffusion-limited aggregation (DLA) of rods into fibers, under the condition that the rods will not move significantly after assembly, as shown in a recent modeling paper [47]. Before activation, a fibrinogen solution is essentially a colloidal solution of noninteracting fibrinogen molecules (particles), which upon activation quickly aggregate into a fibrin network. Diffusion-limited aggregation implies that diffusion is the rate-limiting step with most particle encounters resulting in an aggregation event, whereas reaction-limited aggregation implies that a reaction upon an encounter is the rate-limiting step. Song and Parkinson showed in their DLA modeling work that rods will assemble into fibers with an open, fractal-like cross-section [47], very much resembling the model in Figure 6 of our paper. A similar mechanism, activation-limited aggregation, was used by Curtis et al. to model fibrin fiber assembly [48]. Activation-limited aggregation is based on diffusion-limited aggregation, except that some monomers are active (can aggregate), whereas others are inactive (cannot aggregate). Their models also resulted in a clot with an open, fractal structure, and these results may be transferable to single fiber assembly.

It might be thought that thicker fibers would have a higher bond density, because of prevailing lateral aggregation over linear elongation during the fiber formation process. However, our data show the opposite—thicker fibers have a lower bond density. This implies that explaining fiber thickness by just considering the kinetic rates of longitudinal bond formation versus lateral bond formation is not the correct approach. Rather, it could be that fiber thickness depends on how many fibers, and especially how many branch points, are formed early in the aggregation phase, which then sets the scaffold for the later, lateral aggregation phase. Fiber and branch point formation in the early aggregation phase may only depend on how quickly and to what extent fibrinogen gets converted to fibrin. If most fibrinogen is quickly converted to fibrin, it will form many early protofibrils and small fibers with many branch points; if less fibrinogen is converted, it will form fewer fibrils and fibers with fewer branch points. In the later, lateral aggregation phase, protofibrils are then just added to the outside of the already formed, early scaffold. Fewer early fibers with fewer branch points ultimately result in a mesh with thicker fibers. The number (and quality) of lateral bonds in any given fiber may not so much depend on how fast the lateral bonds form, but on other factors like steric hindrance. These other factors may lower the number and quality of lateral bonds toward the periphery of fibrin fibers.

## 5. Conclusion

The internal structure of fibrin fibers, and especially the packing of protofibrils, has been unclear. In this paper, we tried to gain insights into the internal structure of fibrin fibers by using fluorescence intensity and force (modulus) measurement. In the fluorescence intensity measurement, we found that the light intensity, *I*, of a fiber cross-section depends on fiber diameter, *D*, as *I* ∝ *D*^1.4±0.2^  (*N*_*f*_ ∝ *D*^1.4±0.2^), and the protein density depends on *D* as *ρ*_*p*_ ∝ *D*^−0.6^. In the force (modulus) measurements, we obtained a relationship between modulus and fiber diameter as *Y* ∝ *D*^−1.5^, indicating that the bond density of bonds connecting fibrin subunits (protofibril) together decreases dramatically as fiber diameter increases as *ρ*_*b*_ ∝ *D*^−1.5^. These relationships suggest that protofibrils are densely packed and well connected in the center and become sparse and loosely connected toward the periphery.

## Supplementary Material

Turbidity experiments and additional intensity vs. fiber diameter data.
