# Supplementary material for: Nonuniform Internal Structure of Fibrin Fibers: Protein Density and Bond Density Strongly Decrease with Increasing Diameter
Source: Biomed Res Int. 2017 Oct 10;2017:6385628. doi: 10.1155/2017/6385628 (PMC5654258; doi:10.1155/2017/6385628)
Supplement: Supplementary file 1 — Turbidity experiments and additional intensity vs. fiber diameter data. [file 6385628.f1.pdf]

Nonuniform Internal Structure of Fibrin Fibers: Protein Density and Bond Density Strongly Decrease with Increasing Diameter

Wei Li[1], Justin Sigley[1], Stephen Baker[2], Christine Helms[3], Mary T. Kinney[1], Marlien Pieters[4], Peter H. Brubaker[5] Roger Cubcciotti[6], and Martin Guthold[1]

[1] Department of Physics, Wake Forest University, Winston-Salem, NC 27109

[2] Leeds Institute of Cardiovascular & Metabolic Medicine, The LIGHT Laboratories, University of Leeds, Clarendon Way, Leeds, LS2 9NL, United Kingdom

[3] Department of Physics, University of Richmond, Richmond, VA 23173

[4] Centre of Excellence for Nutrition, North-West University, Potchefstroom, South Africa

[5] Department of Health & Exercise Science, Wake Forest University, Winston-Salem, NC 27109

[6] NanoMedica, LLC, Biotech Place, 575 Patterson Ave, Winston-Salem, NC 27101

Supplementary Information

Turbidity experiments

We performed several turbidity experiments with different ratios of unlabeled fibrinogen to Alexa 546-labeled fibrinogen, at the same buffer conditions as the fluorescence intensity experiments (same salt concentrations and pH, same fibrinogen concentrations). The fibrinogen preparations were the same as the ones used in the experiments of the main text. Unlabeled fibrinogen was from Enzyme Research Laboratories, South Bend, IN, and the Alexa-546-labeled fibrinogen (15 fluorophores per fibrinogen monomer) was from Life technologies, Grand Island, NY.

The following reactions were prepared in the wells of a 96-well microtiter plate.

80 µl **Fibrinogen solution** (1.5 ml/mg Fibrinogen (either the unlabeled fibrinogen, or the 3:1, 1:1 or 1:3 mixtures of unlabeled fibrinogen to Alexa-546-labled fibrinogen), 10 mM Hepes, 140 mM NaCl, pH 7.4)

20 µl **Activation mixture** (thrombin (2.5 units/ml), 25 mM $CaCl_2$, 10 mM Hepes; 140 mM NaCl, pH 7.4)

---

100 µl

The activation mixture was added with a twelve-channel pipette right before the plate was put in the plate reader (took about 5 seconds). Final concentrations in these turbidity reactions were: 1.2 mg/ml fibrinogen, 0.5 units/ml thrombin, 5 mM $CaCl_2$, 10 mM Hepes, 140 mM NaCl, pH 7.4.
The turbidity reactions were performed at 37C.
Experiments were performed in quadruplicate (unlabeled fibrinogen and 3:1 mixture) or duplicate (1:1 mixture and 1:3 mixture) and the averages are shown in Figure S1.
All turbidity data were collected on a BioTek Synergy H1 Hybrid Plate Reader. The data collection program was Gen5.

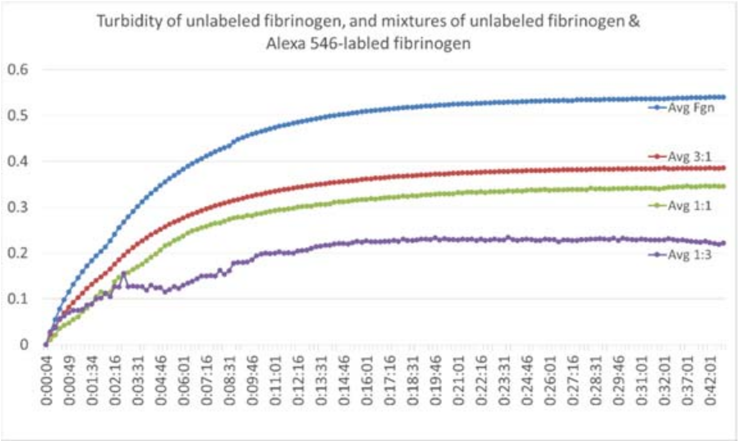

Figure S1. Turbidity curves for unlabeled fibrinogen (blue), and 3:1 (red), 1:1 (green), 1:3 (purple) mixture of (unlabeled fibrinogen):(Alexa-546-labeled fibrinogen). The curves are the averages of quadruplicate measurements (unlabeled fibrinogen and 3:1 mixture), or duplicate measurements (1:1 mixture and 1:3 mixture). Alexa 546-labeled fibrinogen (~15 dye molecules per fibrinogen molecule) affects fibrin fiber formation in a dose-dependent manner. It results in a less step initial slope, and a lower maximum absorption. A less steep slope may indicate slower lateral aggregation and a lower maximum absorption may indicate thinner fibers.

Additional intensity vs. diameter data

- *Dry Electrospun fibrinogen fiber (with Rhodamine 6G)*

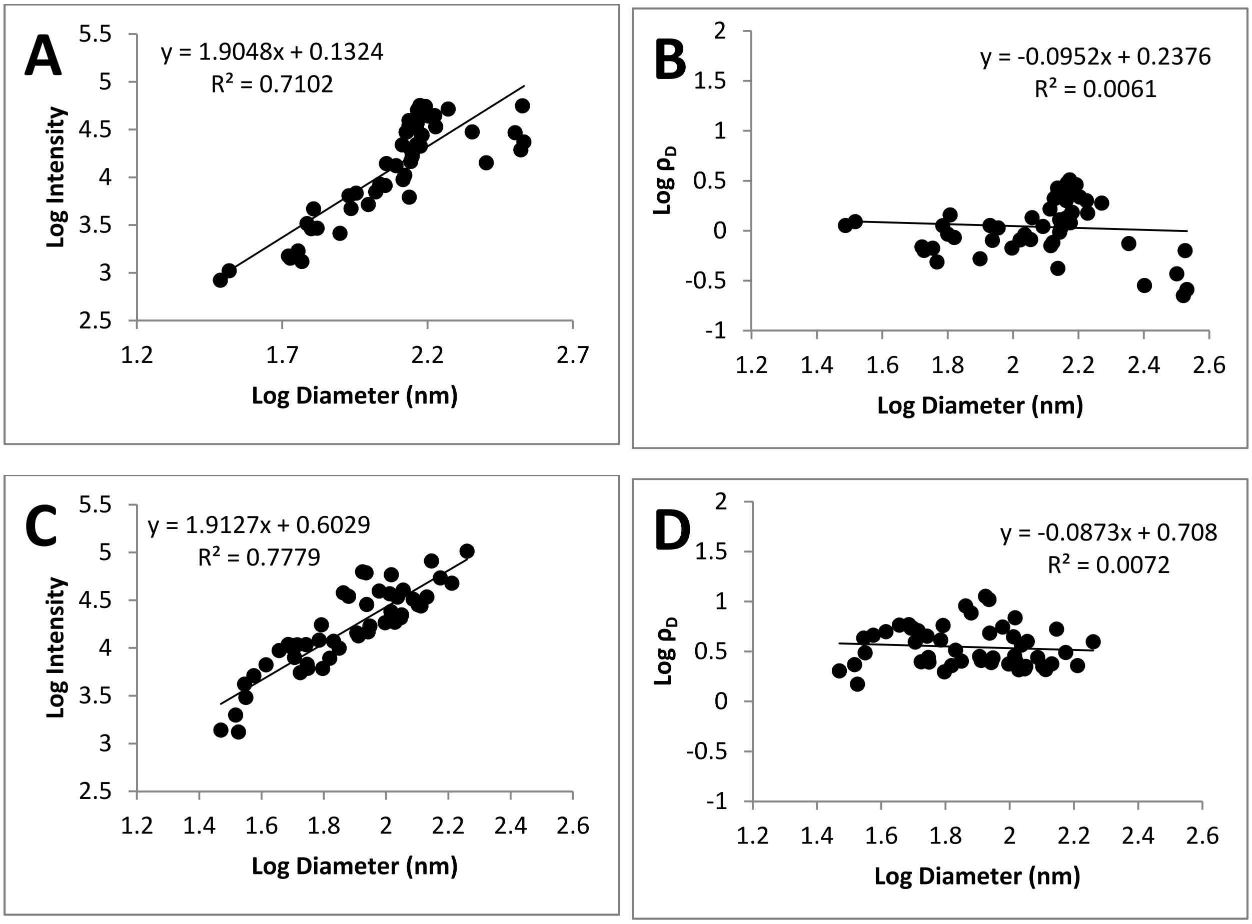

Figure S2. Fluorescence intensity of electrospun fibrinogen fiber cross section as function of diameter. (A) and (C) show the slope of the relationship between fluorescence intensity and fiber diameter on a log-log scale. (B) and (D) show that fibrinogen molecule density (intensity divided by cross-sectional area) is independent of fiber diameter. Each data point represents four measurements.

❖ *Wet fibrin fiber (in fibrin buffer with Alexa 546)*

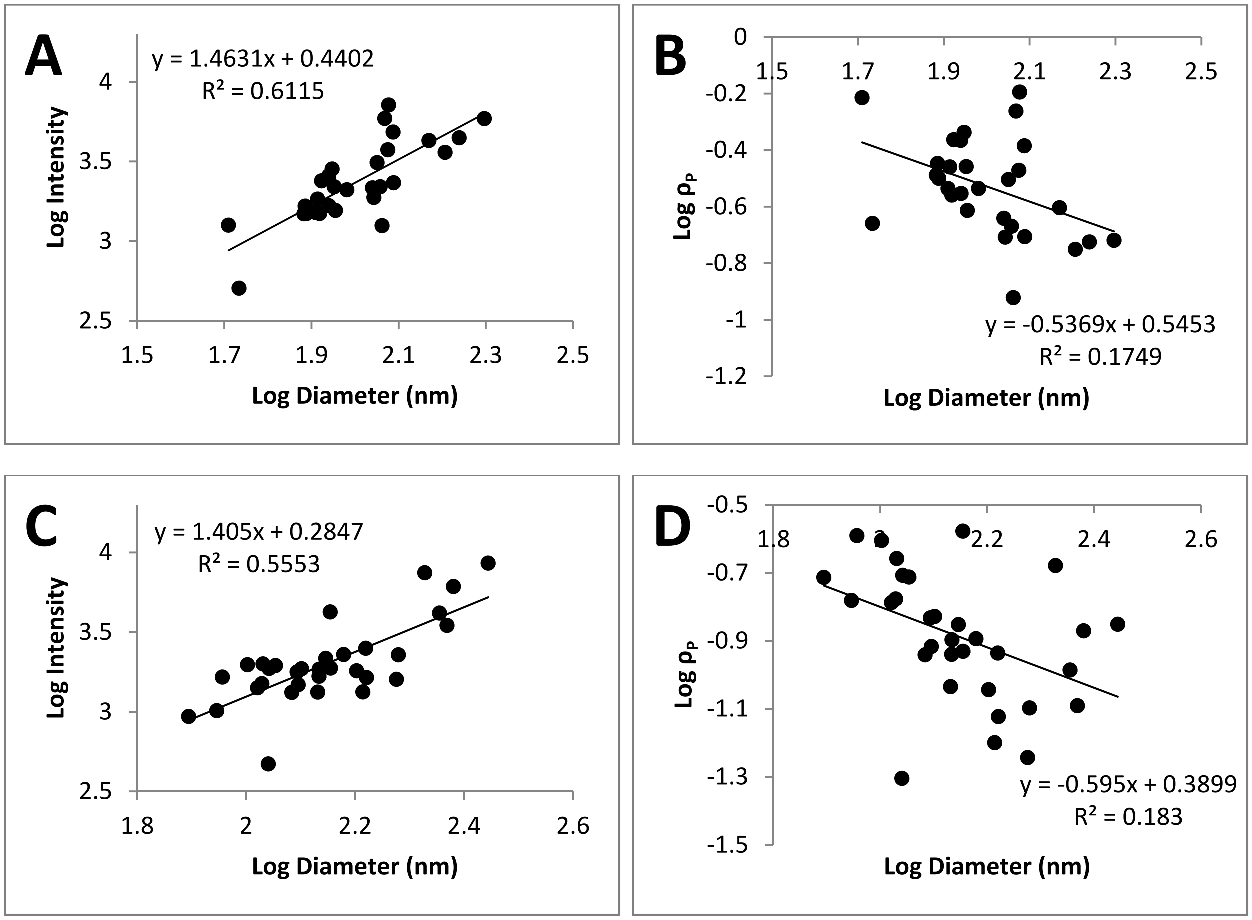

Figure S3. Fluorescence intensity of fibrin fiber (in buffer) cross section as function of diameter. (A) and (C) show the slope of the relationship between fluorescence intensity and fiber diameter on a log-log scale. (B) and (D) show the slope of the relationship between protein density (fluorescence intensity divided by cross-sectional area) and fiber diameter in log-log scale. Each data point represents four measurements.

- *Dry fibrin fiber (with Alexa 546)*

For the dry fibrin fiber (dried with Nitrogen), the averaged slope of plot fiber diameter vs. light intensity is 1.21±0.14 (1.24, 1.17, 1.23).

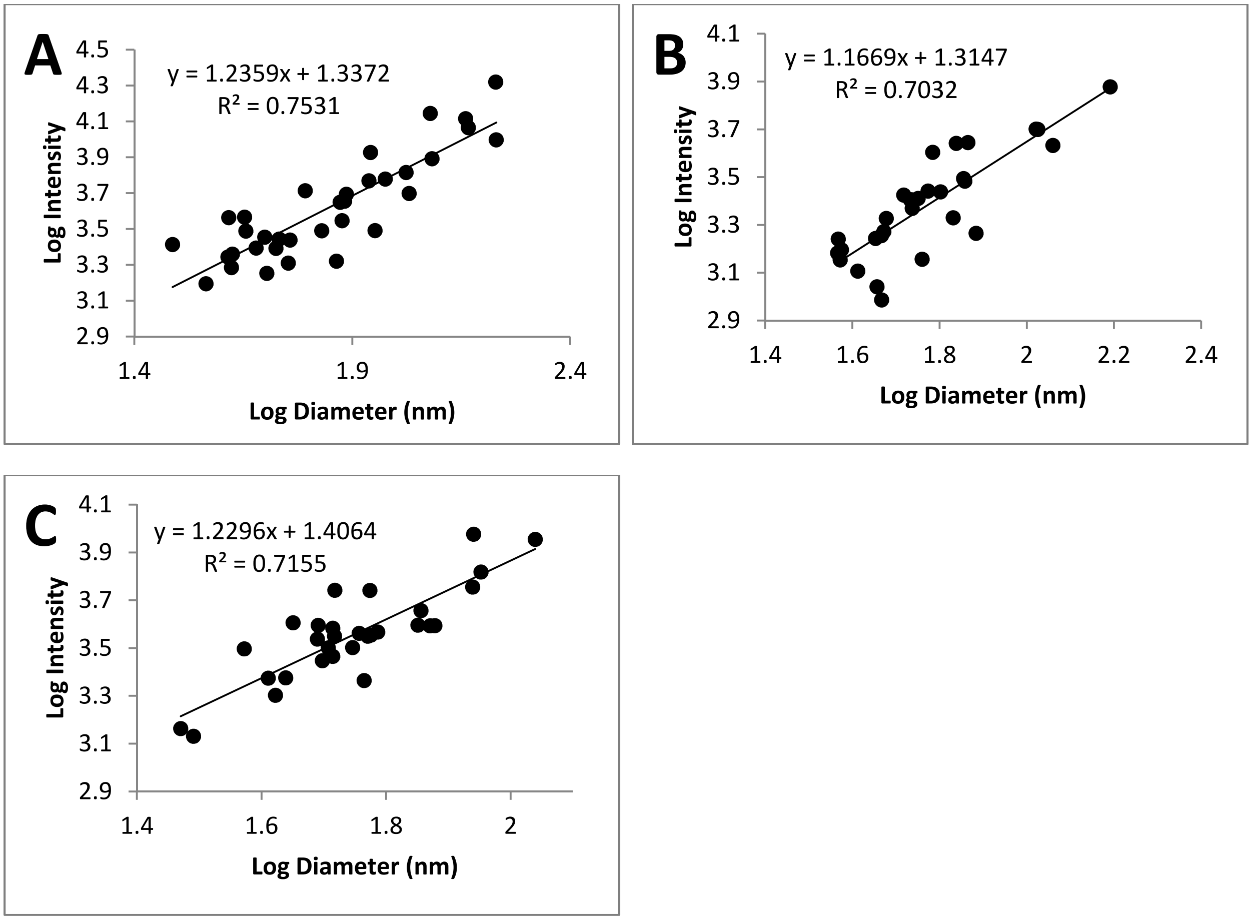

Figure S4. Fluorescence intensity of dry fibrin fiber cross section as function of diameter. (A), (B) and (C) show the slope of the relationship between fluorescence intensity and fiber diameter in log-log scale. Each data point represents four measurements.
